# The Therapeutic Potential of Chemo/Thermotherapy with Magnetoliposomes for Cancer Treatment

**DOI:** 10.3390/pharmaceutics14112443

**Published:** 2022-11-11

**Authors:** Alfonso Toro-Córdova, Monserrat Llaguno-Munive, Rafael Jurado, Patricia Garcia-Lopez

**Affiliations:** 1 Laboratorio de Fármaco-Oncología, Subdirección de Investigación Básica, Instituto Nacional de Cancerología, Cd, Mexico City 14080, Mexico; 2 Departamento de Formulación de Vacunas de mRNA, CerTest Biotec S.L., 50840 Zaragoza, Spain; 3 Laboratorio de Física Médica, Subdirección de Investigación Básica, Instituto Nacional de Cancerología, Cd, Mexico City 14080, Mexico

**Keywords:** magnetoliposomes, cancer, nanotechnology, hyperthermia

## Abstract

Cancer represents a very grave and quickly growing public health problem worldwide. Despite the breakthroughs in treatment and early detection of the disease, an increase is projected in the incidence rate and mortality during the next 30 years. Thus, it is important to develop new treatment strategies and diagnostic tools. One alternative is magnetic hyperthermia, a therapeutic approach that has shown promising results, both as monotherapy and in combination with chemo- and radiotherapy. However, there are still certain limitations and questions with respect to the safety of the systemic administration of magnetic nanoparticles. To deal with these issues, magnetoliposomes were conceived as a new generation of liposomes that incorporate superparamagnetic nanoparticles and oncological pharmaceuticals within their structure. They have the advantage of targeted and selective drug delivery to the diseased organs and tissues. Some of them can avoid the immune response of the host. When exposed to a magnetic field of alternating current, magnetoliposomes produce hyperthermia, which acts synergistically with the released drug. The aim of the present review is to describe the most recent advances in the use of magnetoliposomes and point out what research remains to be done for their application to chemo-thermal therapy in cancer patients.

## 1. Introduction

Cancer represents a very grave and quickly growing public health problem. It deteriorates the quality of life of patients and is the first cause of mortality worldwide. In 2021, there were 20 million new cases and 10 million cancer-related deaths. The highest incidence in men was for lung, prostate, colorectal, stomach, and liver cancer, and in women for breast, colorectal, lung, cervix, and stomach cancer. The countries with low to median income are disproportionately affected by cancer, and they will carry the burden of 70% of cancer-related deaths by the year 2040, according to the World Health Organization (WHO) and the International Atomic Energy Agency (IAEA) [1].

Chemotherapy is one of the pillars of cancer treatment, as are radiotherapy and surgery. Over 100 agents are currently used as antineoplastic drugs, and many more are still under investigation. Unfortunately, the great majority of these drugs lack specificity towards cancer cells. Consequently, they are toxic not only to tumor tissue but also to healthy tissue and organs, especially those with a rapid rate of proliferation (e.g., bone marrow, the gastrointestinal tract, and hair follicles). Moreover, chemotherapy drugs tend to be toxic to organs involved in their metabolism and elimination. The variability in the toxicity of antineoplastic drugs is dependent on their mechanism of action and target.

The lack of selectivity of conventional anticancer drugs has limited the maximum allowable dose and thus impeded their capacity to accumulate sufficiently in the target tissue. Many of the adverse effects are associated with their distribution in healthy tissue. Additional problems of importance are the pharmacokinetic profile, biodistribution, and chemical instability of a drug [2,3].

The physiology of an organism is altered by cancer and other pathological conditions. The altered organism in turn substantially modifies the pharmacokinetics of drugs taken. In the case of neoplasms, solid tumors are similar to an organ in their structure but are more heterogeneous and complex. There is a notable difference in the quantity and composition of the extracellular matrix of a tumor compared to that of healthy tissue [4]. The elevated interstitial fluid pressure caused by these differences hinders the internalization of drugs by a tumor.

Since drugs reach their target site by the systemic route, the pharmacological response of the tumor is influenced directly or indirectly by the vasculature. The blood vessels of tumors are characterized by a greater dilation and quantity of ramifications as well as a lack of orderly growth. Moreover, these vessels are compressed by tumor cell growth, their vascular endothelium has fenestrations, their basal membrane is discontinuous, and their blood flow is disorganized and variable. All the aforementioned factors increase resistance to blood flow and give rise to an abnormal distribution of blood and consequently of nutrients and oxygen in the tissue of a given tumor. Thus, some regions of tumors commonly present hypoxia, acidity, and a distinct metabolic activity, a microenvironment that leads to a lesser accumulation of therapeutic agents [5]. In response to the difficulty of targeting drugs to tumors, the last decade has seen an accelerated development of new highly selective anticancer treatments in order to enhance the therapeutic effect and diminish toxicity.

Among the new breakthroughs in oncological treatment is the use of nanotechnology for the elaboration of liposomal systems as transporters of therapeutic agents [6]. Additionally, these systems are instrumental in the generation of elevated temperatures at the target site (hyperthermia) [7]. The encapsulation of magnetic particles in liposomes, denominated magnetoliposomes, has proven to be advantageous. The purpose is to magnetically guide the liposomes to a tumor and then create hyperthermia with a magnetic field produced by alternating current [8,9]. As a result, the encapsulated drug is released and works synergistically with the elevated temperatures to provoke the death of cancer cells. The progress in the development of liposomes in general and the most recent advances in magnetoliposomes are discussed in the current contribution by reviewing relevant reports.

## 2. Liposomal Nanosystems in Oncology

Liposomes, first described during the 1960s, are defined as spherical vesicles of nanometric size that form when phospholipids or similar amphipathic lipids are hydrated or exposed to an aqueous environment [10]. They are composed of an aqueous nucleus contained within one or more bilayers of natural or synthetic phospholipids.

As systems for the release of drugs, liposomes are often capable of improving the therapeutic index by increasing the concentration of a drug in tumor cells and/or decreasing its toxicity for healthy tissue. Various antineoplastic drugs have been successfully formulated by utilizing liposomes as vectors (Table 1). The versatility of liposome systems is based on the encapsulation of hydrosoluble as well as liposoluble molecules. This has made them ideal for simultaneously transporting two or more elements, among which are drugs, proteins, selective inhibitors, radionuclides, and magnetic nanoparticles. In addition, they have good biocompatibility, low toxicity, and limited immunological effects because they are composed of lipids of natural or semisynthetic origin [11]. A list is herein offered of the liposomal formulations approved for cancer treatment (Table 1).

### 2.1. Toxicity

It has been shown that liposomes are not completely innocuous even though the components utilized in their elaboration are generally considered non-toxic due to their natural and semisynthetic origin. A variety of adverse effects and immunogenic reactions have been reported after the administration of commercial formulations of liposomes [13,14]. Additionally, an increase in the dose of phospholipids can decrease the plasmatic concentration of diverse proteins. Although these proteins have not yet been identified and their biological significance is unknown, such changes are likely to alter homeostasis [15].

### 2.2. Biodistribution and Pharmacokinetics

As a result of the discontinuous vascular endothelium found in a tumor (facilitating the extravasation of liposomes to the interstitial space), liposomes are capable of passively accumulating in a tumor by means of the enhanced permeability and retention (EPR) effect. Also favoring the accumulation and retention of liposomes at this site is the lack of lymphatic drainage in tumor tissue [16] Once in the interstitial space, the drug enters tumor cells by different mechanisms, suggested in one study to include simple diffusion through the cell membrane, and endocytosis [17].

The pharmacokinetic profile and biodistribution is quite different for drugs encapsulated in liposomes compared to their free form. The encapsulated drugs essentially follow the pharmacokinetics and biodistribution of the vector (the liposome). The pharmacokinetic parameters of liposomes depend on the dose and route of administration as well as their physicochemical properties, including size, surface charge, composition of the phospholipidic bilayer, and the modification of the liposomal surface through the binding of distinct compounds [18,19,20,21]. For instance, the volume of distribution of the encapsulated drugs is sharply reduced and the plasmatic concentration is increased because liposomes do not bind to plasmatic proteins. As a consequence, there is a greater concentration of the drug in circulation and better bioavailability.

Conventional liposomes administered intravenously are rapidly recognized by the reticuloendothelial system and removed from circulation, accumulating mainly in organs such as the liver and spleen [22], thus representing a problem if these organs are not the target. One of the most successful strategies for slowing down the elimination of liposomes from circulation consists of modifying their membrane to make it more hydrophilic, which is accomplished by binding polyethylene glycol (PEG) or other polymers to it. The resulting liposomal formulations are known as stealth liposomes (or sterically stabilized liposomes) [23] due to their capacity to evade the immune system. This enables them to deliver a higher percentage of the drug dose to the target tissue compared to conventional liposomes elaborated without stealth technology. Hence, the fundamental characteristic of stealth liposomes is their slow elimination. Their plasma half-life is approximately 2–3 days, and they are still found in plasma one- or two-weeks post-administration.

**Figure 1:**
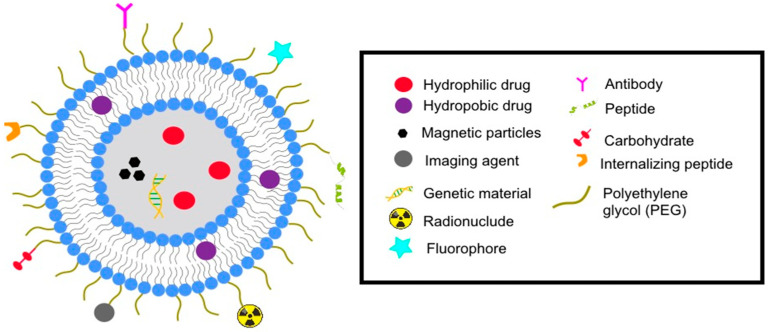
Principal functionalizations of liposomes to establish them as transport systems for drugs.

Other modifications have been made to liposomes with the aim of generating transport systems with more specific purposes, leading to greater therapeutic efficacy. For example, some function as theragnostic agents (therapy + diagnosis) or combine therapies (e.g., radiation and hyperthermia) (Figure 1).

## 3. Oncological Hyperthermia

The therapeutic value of elevating the temperature above normal, of a part or the whole body, to the treatment of a disease has been of interest for thousands of years. This concept known as hyperthermia has become an important focus of cancer research since the discovery of the radiosensitizing action of heat two or three decades ago. Around 4000 scientific articles have been published on inducing hyperthermia for the treatment of cancerous tumors.

Upon being exposed to elevated temperatures (≥41 °C), cells are irreversibly damaged, especially their proteins. Cell damage caused by thermal shock includes the decomposition of the cytoskeleton, denaturation of cytoplasmic proteins, loss of membrane receptors, and damage to membrane integrity. Cells are reportedly more thermosensitive in the S or M phase of the cell cycle [24], at acidic pH, and under nutrient deficiency. These results obtained under controlled conditions in cell cultures suggest that tumor cells are particularly sensitive to hyperthermia, considering their acidic and nutrient-poor condition [25].

The first effect observed with an increase in temperature in tissues is a greater blood flow as a response to dissipating heat. However, the limited possibility of greater blood flow in a tumor hinders the dissipation of heat through this mechanism. As the temperature rises and the exposure time lengthens, edema and vascular lesions may occur [26]. Studies in tumor models confirmed that irreversible heat and cell stress induced by this treatment can lead to programmed cell death (apoptosis) and the release of damage-associated molecular pattern signals relevant for inducing an immunogenic cell death [27]

In addition to the cytotoxicity detected in preclinical models, hyperthermia at temperatures over 42 °C has generated a reduction in tumor blood flow, thus impeding the arrival of oxygen and nutrients. As a consequence, acidosis and damage to the tumor vasculature were found, resulting in the swelling of the endothelium, flow of plasma fluid to the interstitial space, micro-thrombosis due to the activation of hemostasis, and a change in the viscosity of blood cell membranes. At the same time, no damage was exhibited by the vasculature of normal tissue.

Contrarily, tumors treated with moderate hyperthermia (42 °C, easily attained in preclinical models and even in patients) show a greater blood flow and therefore higher levels of oxygen. This can enhance the efficiency of radiotherapy and chemotherapy. The former is more effective with a higher concentration of oxygen, while the latter is more effective with greater blood flow, which increases the probability of the accumulation of a therapeutic agent in a tumor [28,29].

In vitro studies have demonstrated that hyperthermia and chemotherapy act synergically on tumor cells. Maurici et al. (2022) reported that hyperthermia increases the cytotoxic effect of 5-fluorouracil, gemcitabine, and cisplatin in pancreatic cancer cell lines. There are several mechanisms by which hyperthermia could help increase the cytotoxic effect of antineoplastic agents [30]. Moreover, hyperthermia improves the cytotoxicity of various antineoplastic agents by causing changes in the fluidity and stability of the cell membrane and in the membrane potential. It also disrupts transmembrane transport (by altering apoptosis resistance proteins, leading to apoptosis), hinders the proper synthesis of proteins and denaturation of the same, induces the synthesis of thermal shock proteins, damages the synthesis of RNA and DNA and inhibits the enzymes responsible for their repair, and alters the conformation of DNA [31].

The use of hyperthermia as a part of oncology treatment has increased in the last years; however, there is a controversy about its effectivity. Quenet et al. (2021) did not find a significant difference in global survival with the use of hyperthermic intraperitoneal chemotherapy in comparison with the use of cytoreductive surgery only. For that reason, more studies are needed on the use of hyperthermia as an oncology treatment [32].

### 3.1. Techniques for the Application of Hyperthermia

The principal limitation of clinical hyperthermia has been the design of equipment and methodologies to increase the temperature locally and uniformly in tumor mass without affecting the surrounding healthy tissue. In recent years, much research has focused on resolving this problem in order to improve the efficacy of treatment. The principal hyperthermia techniques were local hyperthermia, regional and entire body [33].

Hyperthermia of the entire body has been accomplished in distinct manners, the main one being the exchange of heat to the body from external sources. This occurs with the use of thermal garments or blankets, immersion in a hot water or wax bath, and perfusion of blood at elevated temperatures. Apart from the high risk of systemic toxicity (especially if the technique is combined with the administration of drugs), a limited and temporal response has been observed in patients [34].

Regional perfusion has been utilized to treat well-localized tumors, particularly in the case of sarcomas and melanoma of the extremities. Intracavitary perfusion has been investigated as well and has found high toxicity [35]. With other hyperthermia techniques developed recently, including the majority of clinical applications of hyperthermia, heat is provided by microwave energy, radiofrequency, and ultrasound. These heat sources are considered relatively noninvasive and have the capacity to cause localized hyperthermia. Nevertheless, one of the main disadvantages is that in tumors over 2–5 cm deep, the distribution of energy in the tumor becomes less homogenous and localized and a significant amount of energy is deposited in surrounding healthy tissue, similar to what occurs with radiotherapy [26,36].

To address this situation, new techniques for generating heat have been developed. One such technique is oncothermia, which involves iron oxide nanoparticles together with a magnetic field. Nanoparticles at a certain dose are focused only on the tumor (without binding to surrounding healthy tissue), and they are activated by a radiofrequency modulated at 13.56 MHz induced between two plan-parallel electric condenser plates embracing the tumor area between two electrodes [27]. The modulated radiofrequency is automatically targeted to the malignant tissue, being the path of least impedance. Meanwhile, the surrounding healthy tissue is isolated by its cell membranes, which are charged by an electronic field with a strength of over one million V/m [37].

Iron oxide nanoparticles are an attractive material for biomedicine because it is possible to control their size, and the particles are easily manipulated during synthesis [38]. Additionally, they can be controlled externally by a magnetic field and resonate when the magnetic field is changed (e.g., by alternating current). The energy received is transformed into heat, allowing for the local production of heat in tumor tissue. It has been proposed that iron nanoparticles are capable of selectively increasing the temperature of molecules bound to them or those nearby.

Considering their great capacity to interact with biological systems, these nanoparticles are important elements for the transport of drugs to tumors. After loading iron oxide nanoparticles with anticancer drugs, it is possible to conjugate them with antibodies to achieve binding to specific receptors on the surface of tumor cells. The result is a therapy targeted in a controlled manner by an external magnetic field to obtain a substantial accumulation of the therapeutic agent in the target tumor tissue [39,40].

### 3.2. Magnetic Hyperthermia

**Figure 2:**
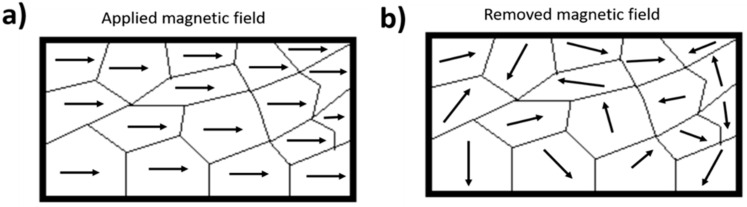
Magnetic behavior in function of magnetic field. (**a**) Superparamagnetic nanomaterial aligns in a similar manner in the direction of the external magnetic field. (**b**) Magnetic moment of the particles returns to its initial orientation.

The heat generated by magnetic nanoparticles in the presence of an alternating magnetic field is focused on tumor tissue. This minimally invasive technique, known as magnetic hyperthermia, aims to locally increase the temperature of tissue. The superparamagnetic nanomaterials are administered to the organism intravenously and targeted to the site of the tumor by means of external magnetic fields. Subsequently, the application of magnetic fields of alternating current make the nanoparticles vibrate and therefore heat is produced [41]. With this external stimulus, the magnetic moments of the superparamagnetic nanomaterial align in a similar manner in the direction of the external magnetic field. When the stimulus is withdrawn, the magnetic moment of the particles returns to its initial orientation and no remnant magnetization is present (Figure 2).

**Figure 3:**
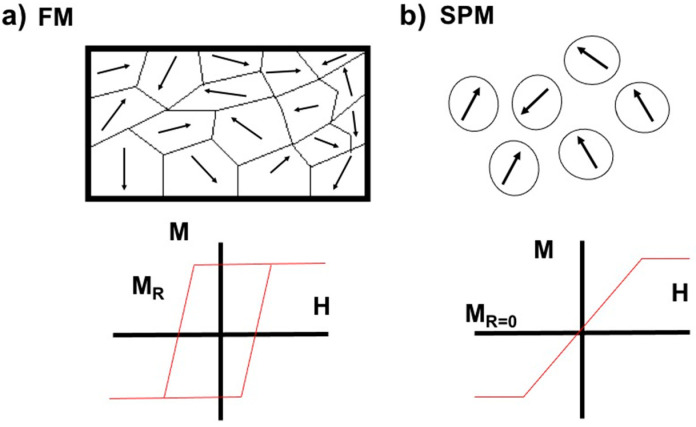
Magnetic behavior as function of the size of nanoparticles. (**a**) Materials made of ferromagnetic nanoparticles (∼50 nm in diameter) have multiple domains and remnant magnetization (M_R_) after withdrawing the magnetic field. (**b**) Materials made of superparamagnetic nanoparticles (10–20 nm in diameter) do not have multiple domains or remnant magnetization after magnetic stimulus is withdrawn. FM (ferromagnetic), SPM (Superparamagnetic). Modified from Krishnan et al., 2010 [41].

During the process of reorientation, energy is released mainly through the processes of Néel relaxation (reorientation of the magnetic moment) and Brown relaxation (rotation of the nanoparticles) [41,42]. The energy released is dispersed as heat to the surrounding tissue, thus generating hyperthermia. This technique avoids the limitations found with other ways of promoting hyperthermia. The nanoparticles provide heat directly to the tumor site, and the quantity of heat that arrives to the surrounding tissue can be controlled. If the size of the nanoparticles is under 20 nm, the magnetic anisotropy energy barrier of a nanoparticle with only one domain is less than the thermal energy. Consequently, the orientation of the magnetic moment of the particle becomes unstable due to thermal agitation. As a result, the particle does not retain permanent magnetization when the external magnetic field is withdrawn, thus becoming a superparamagnetic particle [39,41] (Figure 3). Iron oxide superparamagnetic nanoparticles reach high values of magnetization, making them suitable for certain biomedical applications, such as hyperthermia and the targeting of drugs to tumor tissue.

To generate the necessary magnetic field to increase and control the temperature during the hyperthermia with nanoparticles, there are commercial heat inducers [43], such as the equipment DM 100 system, nB nanoscale Biomagnetics (Zaragoza, Spain) and MagneTherm (NanoTherics) (Warrington, United Kingdom) [44,45,46]. All these instruments provide an accurate control of temperature during hyperthermia in cellular cultures and in tumors generated in laboratory animals.

Among the formulations that have been elaborated with iron oxide magnetic nanoparticles are those conjugated with the peptide CREKA and coated with dextran [47], and a thermosensitive system loaded with 5-fluorouracil (Fe_3_O_4_/PNIPAM/5-Fu@mSiO2-CHI/R6G) [40]. In the latter system, hyperthermia is generated by controlling the magnetic field. An alternating magnetic field employed to produce hyperthermia can excite particles without any limitations on the depth of the tumor. Indeed, this technique has been utilized clinically to treat deep tumors that are not resectable, including glioblastoma [48] or prostate tumors [49].

## 4. Magnetic Nanoparticles

Two iron oxide nanoparticles, magnetite (Fe_3_O_4_) and maghemite (γ-Fe_2_O_3_), are among the most common nanomaterials used to promote hyperthermia through magnetism. They have both been investigated in relation to biomedical applications for diagnostic purposes, serving as contrast agents for magnetic resonance imaging (MRI) [50,51]. One of the main advantages of magnetite is its superior biocompatibility compared to other magnetic materials, given its similarity to the iron present in organisms. Hence, toxic and immunogenic effects are limited. Since it has an elevated value of the magnetic moment, the response of magnetite becomes potent when exposed to external magnetic fields. The size of the particle with the capacity of adopting a superparamagnetic behavior is ∼20–30 nm [52].

Iron oxide nanoparticles (IONPs) are the only nanomaterial approved by the FDA for clinical use. They have low toxicity and are highly biodegradable and biocompatible. These nanoparticles are relatively unstable with low solubility and bioavailability and a strong tendency to aggregate. Additionally, their magnetism is lost to oxidation. Thus, they need to be functionalized, meaning that functional groups with hydrophilic ligands must be added to allow them to be stabilized and transported through the blood without aggregating, disassociating, opsonizing, losing magnetism, or entering into chemical reactions capable of changing their structure and properties. Therefore, iron oxide nanoparticles are coated with polymeric matrices.

Thermosensitive coatings made of nanoparticles have been coupled to drugs, in which case the coatings serve as vectors to target the drugs to tumor cells. This leads to a maximum drug concentration in tumor tissue and a minimum of adverse effects. The hyperthermia generated by these nanoparticles upon exposure to an alternating magnetic field causes death exclusively to cancer cells [53].

Iron oxide nanoparticles are classified as superparamagnetic or ferromagnetic in accordance with their size. The most commonly used in nanomedicine are superparamagnetic nanoparticles, with a diameter of 10–20 nm. They have been employed as contrast agents for MRI studies and as transporters for the controlled release of drugs to tumors. In the latter role, it has been possible to functionalize them with different coatings, including polyacrylic acid [54], dextran [55], polyethylenimine [56], silica [57], carbon [58], gold or silver [39,59].

Iron oxide nanoparticles (IOPNs) are very often synthesized as oleate-coated species to prevent oxidation and loss of magnetism. During loading into liposomes, they are heated at temperatures ranging from 400 to 600 °C and washed to remove the oleate coating and let them to be able for functionalization [44,60,61]. However, for nanoparticles encapsulated in the aqueous core or conjugated to the surface of liposomes, oleate-coating has been the method most frequently used. In this case, it is expected that hydrophobic nanoparticles have a higher affinity to the phospholipid bilayer [62,63]. Martinez-Gonzales et al. reported the synthesis of hydrophobic magnetic nanoparticles which were loaded into the lipid bilayer of the liposome. These magnetoliposomes showed their feasibility to be used as contrast agents for MRI. The contrast was particularly enhanced when hydrophobic nanoparticles were used [64].

### 4.1. Pharmacokinetics and Bioavailability of Magnetic Nanoparticles

The distribution in organs and the pharmacokinetics of iron oxide nanoparticles depend to a great extent on their physicochemical properties, such as size, morphology, surface charge, and the presence or absence of molecules bound to their surface. These properties are capable of substantially changing the behavior of nanoparticles in the organism. Among other experimental variables that can influence the pharmacokinetics and biodistribution of this type of material are the routes of administration and the variations between animal models and humans.

For research on the treatment of cancer, some of the routes of administration most commonly evaluated are intravenous, oral, and intratumoral. The immune system is known to respond rapidly to the presence of free nanoparticles in an attempt to eliminate them from the bloodstream, regardless of the via of administration. The most important mechanism of elimination is the phagocytic activity of cells of the reticuloendothelial system in certain organs, especially the liver and the spleen, where the greatest accumulation of free nanoparticles is found [65,66]. When applied at high doses, these nanoparticles also accumulate in other tissues containing cells of the reticuloendothelial system, including lymph nodes as well as lung, adipose, bone marrow, and brain tissue.

The circulation half-life of nanoparticles varies from a few minutes to various days, depending on their coating. The instability of the size of nanoparticles caused by their aggregation also plays an important role in their rate of clearance from the organism. If nanoparticles have a sufficiently small size (<10 nm), they can be rapidly eliminated in the kidney by filtration [67]. The mechanisms involved in their intracellular metabolism are thought to be very similar to those of ferritin. Accordingly, the particles are degraded and the excess of iron is stored as ferritin or transferrin, entering into the process of natural iron metabolism in the organism [68].

### 4.2. Toxicity of Magnetic Nanoparticles

Iron oxide nanoparticles are generally considered as safe, biocompatible, and nontoxic. For particles without any coating, a lethal dose (LD_50_) of up to 300–600 mg/kg of body weight has been found. For particles stabilized with biocompatible molecules as dextran, the LD_50_ has reached 2000–6000 mg/kg [69]. However, the physicochemical properties of these nanoparticles, like their pharmacokinetics and biodistribution, greatly influence their toxicological profile. Hence, it is necessary to assess the toxicity of each type of formulation.

The majority of synthesized magnetic nanoparticles exert toxicity by means of the production of reactive oxygen species (ROS), which varies in accordance with their physicochemical properties [70]. Moreover, the toxicity of nanoparticles has been shown to increase as their size decreases, owing to a greater surface area that makes them more reactive and more able to penetrate tissues [71]. The greater the variability in the biodistribution for each type of nanoparticle, the more complicated the toxicity studies become. Toxicity is analyzed in vitro based on the evaluation of metabolic activity in cells, while it is examined in experimental animals through biometrics, blood chemistry, histopathological tissue sections, and the monitoring of the general condition and weight of the animals [68]. The molecular mechanisms by which iron oxide nanoparticles exhibit increased cytotoxicity are still being studied. These nanoparticles have been observed to generate an increase in reactive oxygen species, leading to DNA damage and inducing apoptosis [72]. In addition, it has been reported that they present an essential disruption to the immune system stimulating it to recognize tumor cells and increase therapeutic efficacy [73].

In the last years, the use of iron oxide nanoparticles has recently been extensively studied both in vitro and in vivo in different types of cancer, as well as in clinical trials, with NanoTherm^®^ being the first approved therapy in Europe based on iron nanoparticles for the treatment of brain tumors [74]. A clinical trial is currently underway for their use in prostate cancer patients (clinical trials NCT02033447) [75].

The few magnetic nanoparticle formulations approved for clinical use have demonstrated a safe toxicological profile in patients [70,76]. It is still necessary to appraise the possible long-term adverse effects.

## 5. Magnetoliposomes

Given the advantages of the use of liposomes and magnetic nanoparticles in cancer therapy, we can expect that the combination of both could be more effective in cancer treatment. Considering the advantages of liposomes such as biocompatibility, reduced toxicity, biodistribution and stability, this system could be used as a coating for magnetic nanoparticles.

Liposomes capable of incorporating magnetic nanoparticles have colloidal structures, which are formed when IOPNs are surrounded by a bilayer of phospholipids. These structures, denominated magnetoliposomes, were described for the first time in the 1980s by Kiwada et al. (1986) [77], and since then have been extensively studied as a transport system for agents utilized in diagnosis as well as therapy. Their amphiphilic properties allow encapsulation, both hydrophilic (aqueous nucleus) or hydrophobic (inside the lipid bilayer). Depending on the desired application, the magnetic nanoparticles can be encapsulated in the aqueous nucleus, within the phospholipid bilayer or magnetoliposomes based on surface-conjugated nanoparticles [78]. For instance, magnetic particles are preferably encapsulated in the aqueous lumen in the event that liposomes serve as contrast agents for MRI in the diagnosis of cancer [79,80,81].

**Figure 4:**
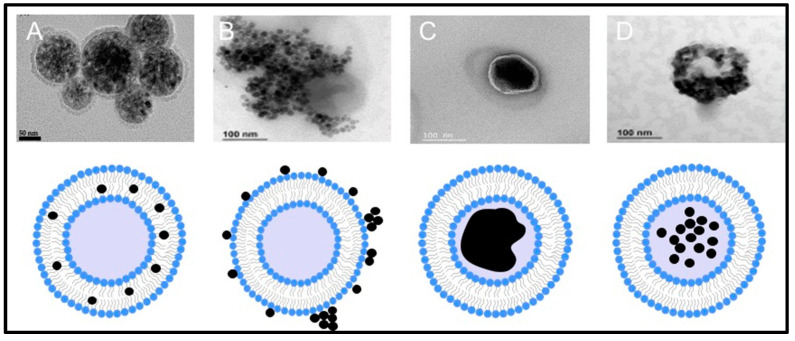
Electron microscopy images (TEM) of the types of magnetoliposomes and schematic representation (not scaled), as examples: (**A**) magnetic nanoparticles within the phospholipid bilayer (partially taken from reference [63], MDPI, Magnetochemistry 2021, 7, 51); (**B**) surface-conjugated nanoparticles (reprinted from reference [9], Copyright © 2020, American Chemical Society, ACS Applied Materials & Interfaces 2020, 12, 4295–4307); (**C**) solid magnetoliposomes (partially taken from reference [43], MDP1, Pharmaceutics 2021, 13); (**D**) magnetic nanoparticles encapsulated in the aqueous nucleus (reprinted from reference [9], Copyright © 2020, American Chemical Society, ACS Applied Materials & Interfaces 2020, 12, 4295–4307).

In the first type (classic) of magnetoliposomes, a center of iron oxide of ∼15 nm in diameter is surrounded by a lipid bilayer. These do not have an aqueous internal cavity, given that this site is occupied only by iron oxide. The second type of magnetoliposomes, developed later, are unilamellar vesicles with a diameter of 100–500 nm. They have many magnetic particles from 1–10 nm in their central aqueous cavity. Usually prepared for extrusion, they are defined as extruded magnetoliposomes. Their principal advantages include the facile modulation of their size and their capacity to contain hydrophilic molecules in the central aqueous space (Figure 4).

The ferromagnetic nanoparticles of iron oxide used in the fabrication of magnetoliposomes have a diameter under 50 nm and should be coated with peptides or other molecules to avoid their aggregation and obtain stable aqueous suspensions. Because they have a relatively small magnetic dipole capable of conferring superparamagnetism, they are designated ultrasmall superparamagnetic iron oxide nanoparticles (USPIOs). When an external alternating magnetic field is applied and then removed, they do not present any remnant magnetism, which constitutes their main characteristic. Consequently, it is not necessary to demagnetize them.

Magnetoliposomes can be modified to bind to immunoglobins G and E and utilized in the diagnosis of hypersensitive reactions in patients with respiratory allergies. They are often used as contrast agents in MRIs due to their capacity to darken the low-intensity regions in the field of interest, thus providing an improved delimitation of the tissues or cells in the image. It is possible to carry out this type of study with classic and extruded magnetoliposomes.

In the field of drug transport, magnetoliposomes have excellent biocompatibility and amphiphilic properties and are easily manipulated to attain the desired variation in size. To increase specificity, they are elaborated with antibodies against ligands expressed on the surface of target cells, such as antigens in renal carcinoma, that are not expressed on healthy cells. To prolong circulation in the blood and avoid the reticuloendothelial system, magnetoliposomes are prepared with PEG chains coupled to the surface to prevent opsonization and elimination by macrophages, making them a type of stealth liposome. Extruded magnetoliposomes are preferable for transporting polar drugs, which can be easily encapsulated in their aqueous cavity. Because the classic form of magnetoliposomes does not have an aqueous cavity, drugs require pre-hydrophobization before being encapsulated.

As drug transport systems, one of the advantages of magnetoliposomes is their capacity to accumulate in tissues, both passively and by the guidance of external magnetic fields to the target site. In 2006, Fortin-Ripoche et al. loaded liposomes with nanocrystals of maghemite and magnetically targeted them to xenografts of prostate cancer developed in nude mice. They then assessed the accumulation of the liposomes through MRI studies. The authors found a 7–8 fold increase in the accumulation of liposomes with, versus without, magnets placed on the skin of the tumors [82]. In 2009, Zhu et al. prepared a formulation of thermosensitive magnetoliposomes loaded with methotrexate and targeted them towards skeletal muscle in mice by means of external magnetic fields [83]. Recently Fortes-Brollo et al. (2020), developed doxorubicin-loaded magnetoliposomes. The liposomes were made of 1,2-Dipalmitoyl-sn-glycerol-3-phosphocholine (DPPC) and iron oxide, controlling the nanoparticle size, surface charge and phase transition temperature, and demonstrating that when the nanoparticles were attached outside the liposome, the membrane integrity was preserved, avoiding the leakage of encapsulated drugs. In addition, the magnetic and heating properties allow them to be used as a drug delivery system, controlling the temperature for drug release by applying an alternating magnetic field. When these magnetoliposomes were evaluated in two cell lines, MDA-MB-231 (breast cancer) and HeLa (cervical cancer), the cell death rate demonstrated that doxorubicin release can be triggered by remote control, using a non-invasive external magnetic field. The authors concluded that, depending on the characteristics of the magnetolipomes, the temperature can be controlled with an external magnetic field and therefore, drug release can be manipulated at the cellular level [9].

**Figure 5:**
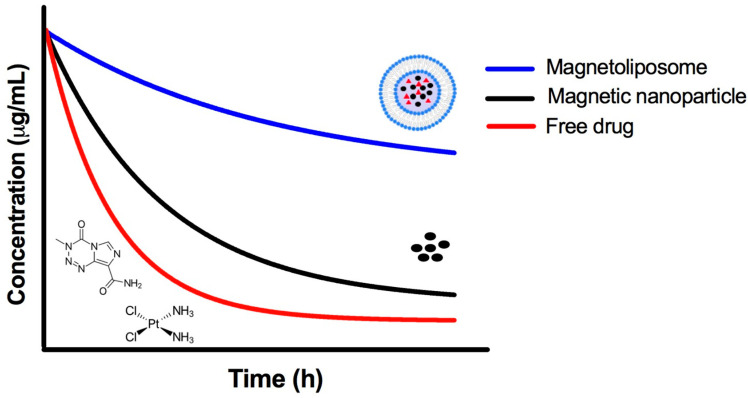
Bioavailability of the magnetoliposome. There is an advantage to the use of nanocarrier systems; when a drug is encapsulated, it is protected from the action of metabolic enzymes and therefore adopts the pharmacokinetics of the nanocarrier systems. There is a prolonged systemic circulation time of the nanocarrier, which implies a greater probability that the drug will reach the target organ or tissue where it is required to be released.

In recent years, the pharmacokinetics and biodistribution of liposomes and magnetoliposomes have been studied, since the pharmacokinetic profile is necessary to predict the effectiveness of these formulations (Figure 5). Earlier, in 2005, Zhang et al. elaborated a formulation of magnetoliposomes loaded with paclitaxel and evaluated them in xenografts of breast cancer in mice, considering their pharmacokinetics, biodistribution, toxicity, and therapeutic efficiency. The results showed a four-fold greater accumulation of paclitaxel in tumor tissue when employing magnetic versus non-magnetic liposomes. Twenty-four hours after i.v. administration, there was an almost 30-fold greater accumulation of paclitaxel with magnetoliposomes versus the conventional formulation of paclitaxel. Compared to non-magnetic liposomes, moreover, magnetoliposomes led to a significant decline in both tumor growth and the uptake of the drug in the liver and spleen [84].

**Figure 6:**
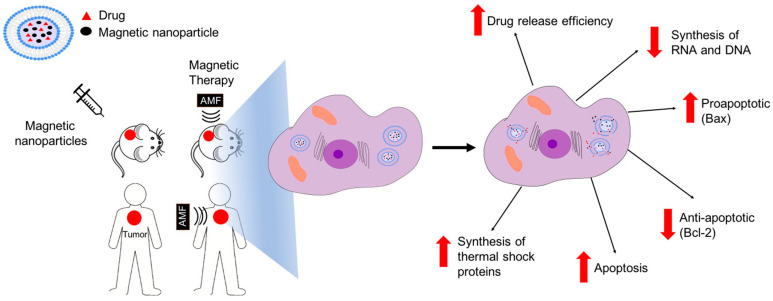
Synergism between magnetic therapy and chemotherapy. The presence of magnetoliposomes enables increased temperatures in local tissues to increase the effect of antitumor drugs and increase apoptosis. There are several mechanisms by which magnetic hyperthermy could act synergically with chemotherapy on tumor cells. Hyperthermia could cause changes in the fluidity and stability of the cell membrane. It also could induce the synthesis of thermal shock proteins, cause damage in the synthesis of RNA and DNA, increase drug release, and alter apoptosis resistance proteins, leading to an increase in apoptosis. Despite significant efforts having been made in the in vitro and in vivo studies of magnetoliposomes, clinical trials have not yet been performed.

The third use of magnetoliposomes is to combine chemotherapy treatment with magnetic hyperthermia. Synergism is known to exist between hyperthermia and the conventional treatments of chemotherapy and radiotherapy [34,85] Figure 6. Once at the target site, it is possible to induce vibration in magnetoliposomes by means of an external alternating magnetic field, thus producing hyperthermia. An example of this type of technology is the loading of magnetoliposomes with doxorubicin, carried out by Babincová et al. (2018) [86].

Another application of magnetoliposomes is in the controlled release of the encapsulated agents. In these cases, hydrophobic nanoparticles are generally employed, being embedded in the phospholipid bilayer of the liposomes. Diverse polymers are coupled to the nanoparticles to make them thermosensitive. As a consequence, they break open at a certain temperature and release the encapsulated drug. The drugs encapsulated in magnetoliposomes can also be released by utilizing magnetic fields of alternating current that create heat and cause the magnetic nanoparticles to modify the permeability of the lipid membrane in such a manner that a greater amount of the drug passes through it [87]. In the majority of cases, the release of the drug is associated with the magnetocaloric effect of the nanoparticles, leading to a phase transition of the lipids of the membrane that does not compromise the membrane structure and integrity [88,89]. This type of liposome has given very good results for the treatment of cancer in preclinical models, yielding a sharp rise in cell death compared to the groups treated with conventional drugs [86,90,91].

To date, diverse formulations of magnetoliposomes loaded with antineoplastic drugs have been developed and evaluated in preclinical tests. Cardoso et al. (2022) elaborated a magnetic nanosystem loaded with doxorubicin, finding an enhanced release of the drug in conditions of hyperthermia and in an acidic environment [92]. Hence, the release of the drug should occur more easily in tumors. It has been reported that magnetic particles could enhance liposome internalization in cancer cells. Redolfi et al. (2020) reported that a stable Dox-Magnetoliposome formulation reduced about 80% cell viability on HepG2 after 72 h of inoculation [93].

In 2010, a formulation of thermosensitive magnetoliposomes loaded with doxorubicin and targeted to folate receptors was prepared by Pradhan et al. (2010). It was examined in vitro in HeLa (cervix cancer) and KB cells in the presence of a fixed magnetic field (a magnet), exhibiting a greater uptake of doxorubicin in cells compared to that found with non-magnetic liposomes. Magnetic hyperthermia reached temperatures of 42–43 °C and was synergistic with the formulation in producing cytotoxicity [94]. In the same year, Yoshida et al. (2010) developed and evaluated a magnetoliposome formulation loaded with docetaxel in xenografts of gastric cancer in mice. To assess the effect of chemo- and thermotherapy, liposomes were directly injected into the tumor and submitted to a magnetic field generated by an alternating current. The formulation reached a temperature of 42–43 °C, which was maintained for 30 min on the surface of the tumors. There was a significantly smaller tumor volume and a significantly higher survival rate of the mice subjected to the combination of chemotherapy and hyperthermia than of those administered either the liposomes loaded only with magnetite or the conventional docetaxel treatment [95]. In other studies, Folic acid-conjugated 17-AAG (17-allylamino-17-demethoxigendanamycin) and DOX-Fe_3_O_4_ magnetic thermosensitive liposomes in combination with an alternating magnetic field for heating could achieve a synergistic anti-tumor effect of chemotherapy and heat treatment [96,97].

Another formulation of magnetoliposomes loaded with curcumin was elaborated and evaluated by Hardiansyah et al. (2017). The authors quantified the capacity of the liposomes to release the drug and the efficiency of the treatment in combination with chemotherapy and hyperthermia. Hyperthermia was induced by a magnetic field. The results showed a significant increase in the release of the drug when the formulation reached 45 °C and synergism between hyperthermia and the drug in producing cytotoxicity on MCF-7 cells [98].

During the last decade various preclinical tests and clinical trials have demonstrated that hyperthermia in combination with cisplatin and other platinum analogs can have a synergistic effect [85,99,100,101,102]. In 2018, our group developed a liposomal system with cisplatin encapsulated in the aqueous center and magnetite nanoparticles embedded in the liposome membrane. These magnetoliposomes were found to cause enhanced cell death through apoptosis. According to the pharmacokinetic data, there was a significant 100-fold increase in the bioavailability of the liposomal formulation and a six-fold increase in its half-life compared to free cisplatin (not encapsulated). In both cases, the route of administration was intravenous [103].

Using a target for cancer cell receptors in magnetoliposomes is a strategy that has been studied. Cintra et al. (2022) reported that folate-target magnetoliposomes loaded with doxorubicin were more cytotoxic for folate receptor overexpression cells [46]. In recent years, the research on magnetoliposomes has increased (Table 2). Although magnetoliposomes have shown positive results, relatively few reports exist on preclinical applications. Hence, these systems should be studied in animal models in the next few years to evaluate their efficacy, pharmacokinetics, and toxicity. The following step would be clinical trials on patients with cancer.

## 6. Conclusions

Great progress has been made in the use of nanotechnology to elaborate magnetoliposomes as systems of transport and release of oncological agents. However, there is still work to be done to make these advances materialize into more effective anticancer treatments. Future studies are necessary to design new and better magnetic nanoparticles that assure heating efficiencies, in specific areas of tumors or deeper tumors, under the application of alternating magnetic fields. Furthermore, novel technologies and materials for thermosensitive magnetoliposomes must be developed to achieve a good magnetic targeting effect, that stimulates heat and thus guarantees a better on-demand drug release and their internalization into tumor cells, reducing drug distribution in normal tissue.

The application of two or more drugs is a common practice used in cancer treatment to achieve synergistic effects. However, adverse effects may also be potentiated in these patients; therefore, the development of magnetoliposomes that combine magnetic hyperthermia with two or more anticancer drugs will represent an important advance for the benefit of cancer patients by increasing the efficacy of oncological drugs and reducing their adverse effects.

Another area of interest in the topic of magnetoliposomes is their diagnostic application, through functionalization with some radiopharmaceuticals, which must continue to be explored to improve magnetic resonance imaging techniques or to develop new diagnostic methods.

Given the implications that the use of magnetoliposomes will have on health, it is important to support the safety and efficacy of these systems through pharmacokinetic and bioavailability studies. To date, the scarcity of information on preclinical pharmacokinetics indicates the need for future research that guarantees an increase in the circulation time of magnetoliposomes in the organism, in addition to ensuring their interactions with tumor cells, leading to significant improvements in the clinical outcome.

## Figures and Tables

**Table 1:** Liposomal formulations used clinically.

Formulation Name (Year of Approval)	Administration Route	Active Agent	Indications	Company
Doxil (1995)	i.v.	Doxorubicin	Ovarian and breast cancer, Kaposi’s sarcoma	Sequus Pharmaceuticals
DaunoXome (1996)	i.v.	Daunorubicin	Kaposi’s sarcoma	NeXstar Pharmaceuticals
Depocyt (1999)	Spinal	Cytarabine/Ara-C	Neoplastic meningitis	Sky Pharma, Inc.
Myocet (2000)	i.v.	Doxorubicin	Metastatic breast cancer	Elan Pharmaceuticals
Mepact (2004)	i.v.	Mifamurtide	Non-metastatic osteosarcoma	Takeda Pharmaceuticals Limited
Marquibo (2012)	i.v.	Vincristine	Acute lymphoblastic leukemia	Talon Therapeutics, Inc.
Onivyde (2015)	i.v.	Irinotecan	Metastatic pancreatic adenocarcinoma	Merrimack Pharmaceuticals Inc.

Modified from Bulbake et al., 2017 [12].

**Table 2:** In vitro and preclinic assays of magnetoliposomes for cancer treatment.

Drug	Magnetoliposome	Cancer Type	Source
Doxorubicin	γ-Fe_2_O_3_	Breast cancer	Liu et al., 2022 [104]
Doxorubicin	Ca0.25Mg0.75Fe_2_O_4_	Sarcoma, breast cancer	Cardoso et al., 2022 [92]
7-[4-(pyridin-2-yl)-1H-1,2,3-triazol-1yl]thieno [3,2-b]pyridine	MnFe_2_O_4_	Cervical carcinoma Breast adenocarcinoma Non-small cell lung carcinoma Hepatocellular carcinoma	Lopes et al., 2022 [105]
Doxorubicin	MnFe_2_O_4_	Tumors overexpressing folate receptors as myelogenous leukemia	Cintra et al., 2022 [46]
Doxorubicin	Ca0.25Mg0.75Fe_2_O_4_	Sarcoma, breast cancer	Cardoso et al., 2021 [43]
17-allylamino-17-demethoxygeldanamycin	Fe_3_O_4_	Hepatoma	An et al., 2021 [106]
Doxorubicin	Fe_3_O_4_	Breast cancer	Ansari et al., 2022 [107]
Curcumin	Ca0.25Mg0.75Fe_2_O_4_	Head and neck, liver, pancreas, colon, prostate, ovary and skin	Cardoso et al., 2020 [60]
Doxorubicin	-DMSA coated-Iron nanoparticles -APS coated-Iron nanoparticles -Oleic acid coated-iron nanoparticles	Breast cancer	Fortes Brollo et al., 2020 [9]
Gemcitabine Paclitaxel	citric acid coated-Fe_3_O_4_ nanoparticles	Invasive breast carcinoma (MGSO-3)	Ribeiro et al., 2020 [108]
Oxaliplatin	MamC protein-Fe_3_O_4_	Colon cancer	Garcia-Pinel et al., 2020 [109]
Betulinic Acid	Fe_3_O_4_	Breast adenocarcinoma	Farcas et al., 2020 [44]
Thienopyridine derivatives	CaFe_2_O_4_	Breast cancer	Pereira et al., 2019 [110]
Doxorrubicin	Fe_3_O_4_	Breast adenocarcinoma	Szuplewska (2019) [111]
Curcumin	MgFe_2_O_4_	Head and neck, liver, pancreas, colon, prostate, ovary and skin	Cardoso et al., 2018 [61]
Anti-CD90+	Fe_3_O_4_	Cancer stem cells	Yang et al., 2016 [112]
Doxorubicin	Fe_3_O_4_	Breast cancer	Liao et al., 2011 [113]
Doxorrubicin	Fe_3_O_4_	Hepatocellular carcinoma	Chen 2014 [114]
Paclitaxel	Fe_3_O_4_	Cervical adenocarcinoma	Liu 2012 [115]

## Data Availability

Not applicable.
